# Low E-cadherin expression in bladder cancer at the transcriptional and protein level provides prognostic information

**DOI:** 10.1054/bjoc.2000.1233

**Published:** 2000-06-15

**Authors:** Z Popov, S Gil-Diez de Medina, M-A Lefrere-Belda, A Hoznek, S Bastuji-Garin, C C Abbou, J P Thiery, F Radvanyi, D K Chopin

**Affiliations:** Service d’Urologie, Centre de Recherches Chirurgicales, EMI-INSERM 99–09 Département de Santé Publique et de Pathologie, Hôpital Henri Mondor, 51 Avenue du Maréchal De Lattre de Tassigny, Créteil Cedex, 94010, France; UMR 144, CNRS/Institut Curie, 26 Rue d’Ulm, Paris Cedex 05, 75248, France; Urology Clinic, Medical Faculty, Sts. Cyril and Methodius University, 17 Vodnjanska st., Skopje, 91000, Macedonia

**Keywords:** bladder carcinoma, E-cadherin, markers, prognosis, gene expression, cell adhesion

## Abstract

We studied E-cadherin down-regulation at the protein level in frozen sections of 111 bladder tumours and 13 normal bladder specimens by means of immunohistochemistry, and at the mRNA level by semi-quantitative RT-PCR in 40 of the same tumours. Results indicate that E-cadherin expression detected by immunohistochemistry correlated with both stage and grade (*P*< 0.0001 and *P*< 0.001, respectively). Analysis of recurrence, progression and survival over a mean period of 36 months after surgery in the entire cohort showed that abnormal E-cadherin immunoreactivity correlated strongly with poor outcome (log-rank test: *P* = 0.001, *P* = 0.0001 and *P* = 0.0003, respectively). In multistep logistic regression analysis, only E-cadherin status and stage had significant additional prognostic value (*P* = 0.008 and OR = 0.2;*P* = 0.03 and OR = 3.6, respectively). Survival estimates derived from RT-PCR transcript quantification differed significantly for low and high expression (log-rank test: *P* = 0.0006). These results suggest that the alteration occurs at the transcriptional level and support the clinical and biological relevance of cell adhesion molecules in bladder cancer. © 2000 Cancer Research Campaign


*British Journal of Cancer* (2000) **83**(2), 209–214
© 2000 Cancer Research Campaign
doi: 10.1054/ bjoc.2000.1233, available online at http://www.idealibrary.com on IDEAL®

### Low E-cadherin expression in bladder cancer at the transcriptional and protein level provides prognostic information

**Z Popov[1,3], S Gil-Diez de Medina[1,2], M-A Lefrere-Belda[1], A Hoznek[1], S Bastuji-Garin[1], CC Abbou[1], JP Thiery[2], F Radvanyi[2] and DK Chopin[1]**

[1]Service d'Urologie, Centre de Recherches Chirurgicales, EMI-INSERM 99–09 Département de Santé Publique et de Pathologie, Hôpital Henri Mondor, 51 Avenue du Maréchal De Lattre de Tassigny, 94010 Créteil Cedex, France; [2]UMR 144, CNRS/Institut Curie, 26 Rue d'Ulm, 75248 Paris Cedex 05, France; [2]Urology Clinic, Medical Faculty, Sts. Cyril and Methodius University, 17 Vodnjanska st., 91000 Skopje, Macedonia

**Summary** We studied E-cadherin down-regulation at the protein level in frozen sections of 111 bladder tumours and 13 normal bladder specimens by means of immunohistochemistry, and at the mRNA level by semi-quantitative RT-PCR in 40 of the same tumours. Results indicate that E-cadherin expression detected by immunohistochemistry correlated with both stage and grade ($P < 0.0001$ and $P < 0.001$, respectively). Analysis of recurrence, progression and survival over a mean period of 36 months after surgery in the entire cohort showed that abnormal E-cadherin immunoreactivity correlated strongly with poor outcome (log-rank test: $P = 0.001$, $P = 0.0001$ and $P = 0.0003$, respectively). In multistep logistic regression analysis, only E-cadherin status and stage had significant additional prognostic value ($P = 0.008$ and OR = 0.2; $P = 0.03$ and OR = 3.6, respectively). Survival estimates derived from RT-PCR transcript quantification differed significantly for low and high expression (log-rank test: $P = 0.0006$). These results suggest that the alteration occurs at the transcriptional level and support the clinical and biological relevance of cell adhesion molecules in bladder cancer. © 2000 Cancer Research Campaign

**Keywords**: bladder carcinoma; E-cadherin; markers; prognosis; gene expression; cell adhesion

Invasive behaviour is a key difference between benign and malignant tumour cells. Acquisition of invasive potential by malignant cells results from an accumulation of characteristics including increased cell motility, secretion of proteolytic enzymes, and alterations of cell–substrate and cell–cell adhesion (Liotta et al, 1991).

Bladder cancer shows a variety of biological behaviours, but acquisition of metastatic capability is the most relevant to clinical considerations. The initial step in local invasion by malignant epithelial tumours is cell detachment from the epithelial sheet.

Decreased cell–cell adhesiveness favours detachment of tumour cells and may play a role in the early steps of the metastatic process. Although cell–cell adhesion is a complex mechanism involving at least four families of adhesion molecules (the integrin, immunoglobulin, selectin and cadherin families), several lines of evidence indicate that E-cadherin-mediated adhesiveness is crucial for epithelial integrity (Takeichi, 1991). Cadherins are functionally related transmembrane glycoproteins responsible for the $Ca^{2+}$ dependent cell–cell adhesion mechanism that underlies the mutual association of vertebrate cells. E-cadherin molecules, which are mainly found in epithelial tissues, contribute to organogenesis and morphogenesis (Shimoyama et al, 1989).

The causal relationship between decreased E-cadherin expression and acquisition of invasive capacity has recently received strong support: modulation of E-cadherin function in several tumour cell lines by the use of blocking antibodies or transfection has revealed an invasion-suppressor role of E-cadherin-mediated cell–cell adhesion in vitro (Behrens et al, 1989; Frixen et al, 1991; Vleminckx et al, 1991). These and other experimental observations suggest that E-cadherin counters tumour spread. E-cadherin has also been examined in a number of human epithelial tumours, such as ductal carcinomas of the mammary gland (Shiozaki et al, 1991), prostate carcinomas (Umbas et al, 1992), and bladder carcinomas (Bringuier et al, 1993; Lipponen and Eskelinen, 1995; Otto et al, 1997). In general, E-cadherin is present in differentiated tumours, whereas expression is lost to variable extents in dedifferentiated, aggressively growing tumours of the same cellular origin, suggesting that the negative relationship between local invasiveness and E-cadherin expression also holds for cancer cells in vivo.

There is still a paucity of markers for bladder tumour aggressiveness, and identification of the molecular steps associated with acquisition of metastatic potential would serve as a basis for diagnostic methods with predictive value (Raghavan et al, 1990). Decreased E-cadherin expression in the bladder has been linked to a loss of differentiation (Frixen et al, 1991; Vleminckx et al, 1991; Behrens, 1994) and tumour aggressiveness (Takeichi, 1993; Rebel et al, 1994) but few studies have compared its predictive value with that of clinical and pathological parameters. Results on the independent clinical value of E-cadherin immunostaining are controversial (Bringuier et al, 1993; Lipponen and Eskelinen, 1995; Otto et al, 1997). E-cadherin immunostaining has been scored arbitrarily as homogenous vs heterogeneous-negative. The mechanism of E-cadherin down-regulation is not fully understood, but it is clear that mutation or deletion of the E-cadherin gene is not the main underlying mechanism in bladder cancer (Knowles et al, 1994; Giroldi et al, 1994). In addition Giroldi et al, (1999) suggested that coregulation of the expression of genes encoding

*Received 16 August 1999*
*Revised 24 February 2000*
*Accepted 7 March 2000*

*Correspondence to:* DK Chopin, Groupe d'Etude des Tumeurs Urologiques, Centre de Recherches Chirurgicales CHU Henri Mondor, 8 rue du Général Sarrail, 94010 Créteil Cedex, France different members of classical cadherins (-E, -P, -N cadherin) occurs during tumour progression and that expression of some catenins is also coordinated with loss of cadherin expression.

To obtain further information on the clinical utility of E-cadherin expression in bladder cancer, we studied this phenomenon at the protein level by means of immunohistochemistry (IHC) and at the mRNA level by semi-quantitative RT-PCR. The protein and mRNA expression was compared to histological parameters for its predictive value as regards to recurrence, progression and survival. We found a significant prognostic value of immunohistochemical staining and the level of RT-PCR transcripts.

### MATERIALS AND METHODS

### Clinical data

Pathological and clinical data are summarized in Table 1. Patients included in this study were recruited between October 1987 and December 1993. They were newly diagnosed cases with a tumour volume sufficient to allow an immediate frozen aliquot. Tumour volumes were measured using an endoscopic loop as ruler. Macroscopic findings from cystoscopies or cystectomies were reported on a scheme including tumour shape (papillary or solid) and size. Patients included were those treated with curative intent by senior urologists as follows: low-risk superficial TCC with transurethral resection (TUR) and follow-up; high-risk superficial TCC with TUR and bacille Calmette-Guerin intravesical instillations. Radical cystectomy was performed for invasive disease or high-risk superficial TCC for which conservative measures failed. None of the patients included in this study had received preoperative radiation or chemotherapy.

Criteria for recurrence and progression were adapted from Herr et al (1989). Recurrence was defined as occurrence of TCC of any stage or grade. Progression was defined as death due to TCC or appearance of distant lymph node or organ metastasis. In case of tumours initially classified as pT2 or higher, clinical recurrence after radical cystectomy was considered progression. In case of tumours initially classified as pTa–pT1, progression was defined as invasion at least to muscle (pT2) or lesion refractory to TUR and intravesical therapy, necessitating a change to more radical treatment (i.e. cystectomy). Disease-specific survival curves were derived taking death from urothelial carcinoma as end-point.

### Immunohistochemistry

Frozen sections (5 μm) from 111 bladder tumours and 13 normal bladder specimens were fixed with 4% paraformaldehyde and analysed by using the mouse monoclonal antibody HECD-1 (Takara Biomedical, Kyoto, Japan) against E-cadherin at 7 μg $ml^{-1}$ dilution and a standard avidin–biotin immunoperoxidase complexes detection system, as according to the manufacturer's protocol (Vectastain Elite ABC kit, Vector Laboratories Inc., Burlingame, CA, USA). E-cadherin expression was scored according to Shiozaki et al, 1991 and Bringuier et al, 1993, who classified tumours as normal if staining was similar to that of normal urothelium. Abnormal tumours were defined as those giving negative (complete absence of immunoreactivity) or heterogeneous staining (positive and negative areas).

**Table 1** Summary of pathological and clinical data. Characteristics of 111 patients with TCC of the bladder, according to age, sex ratio, histologic type and grade (WHO classification) of disease, stage (UICC TNM classification), multifocality, growth pattern and tumour size

| | Number of patients | % |
|---|---|---|
| Male | 92 | 82.9 |
| Female | 19 | 17.1 |
| TCC | 111 | 100 |
| Ta | 15 | 13.5 |
| T1 | 45 | 40.6 |
| T2 | 20 | 18.0 |
| T3 | 22 | 19.8 |
| T4 | 9 | 8.1 |
| G1 | 29 | 26.2 |
| G2 | 32 | 28.8 |
| G3 | 50 | 45.0 |
| N0 | 106 | 95.5 |
| N+ | 5 | 4.5 |
| M0 | 111 | 100 |
| No of tumours: | | |
| Unifocal | 69 | 62.2 |
| Multifocal | 42 | 37.8 |
| Shape of tumours: | | |
| Papillary | 67 | 60.4 |
| Non papillary | 44 | 39.6 |
| Size of tumours in mm, mean (range) | 40 | (3–90) |
| Age in years, mean (range) | 65 | (29–91) |

### RT-PCR assay

RNA was extracted from five normal bladder specimens and 40 TCCs of the bladder (21 Ta/T1; 19 T2/T4; 9 G1, 15 G2 and 16 G3), according to Chirgwin et al (1979), using 4 M guanidinium thiocyanate extraction and cesium chloride ultracentrifugation. The amount of E-cadherin mRNA was determined by semi-quantitative RT-PCR by comparison with an internal control, a ubiquitous transcription factor TBP or GADPH as previously reported (Gil-Diez de Medina et al, 1997). Twenty-two cycles were used for the co-amplification of E-cadherin and TBP.

The primers sequences of TBP and GADPH are as described by Gil-Diez de Medina et al (1997). The primers sequences of E-cadherin are: GACCAAGTGACCACCTTAGA and GCAGGA-ATTTGCAATCCTGCT (5′ to 3′ direction). After gel electrophoresis, the PCR-amplified products were quantified with a Molecular Dynamics 300 PhosphorImager (Sunnyvale, CA, USA).

### Statistical analysis

E-cadherin expression and clinical and pathological parameters were compared in patients with and without recurrence, progression or death from TCC. Means of continuous variables were compared by analysis of variance (multiple groups) and Student's *t*-test (two groups). Frequency distributions were tested by the chi-squared method. Recurrence and progression-free intervals (in person-months) were defined from the date of initial staging (and E-cadherin expression assay) and the date of recurrence or disease progression. Mean follow-up was 36 months, and ranged from 10–136 months. The expression of the E-cadherin transcript was quantified in arbitrary units comparing, using a PhosphorImager, the expression of E-cadherin to the internal control. The cut-off value of E-cadherin expression for low and high expression was defined by 30% of the average values found in normal urothelium.

*© 2000 Cancer Research Campaign*

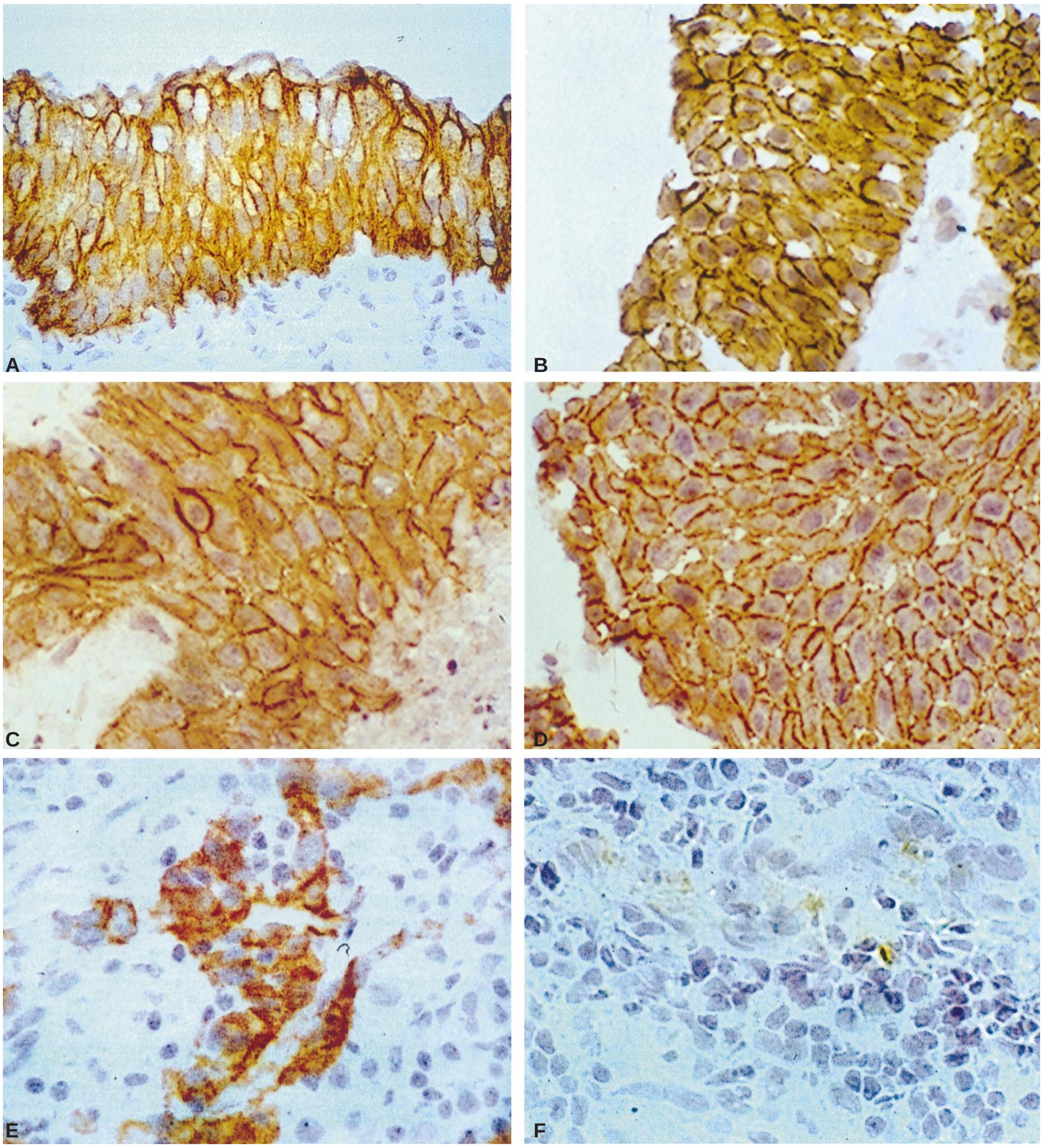

**Figure 1** Representative immunostaining of E-cadherin protein (magnification × 400) (**A**) In normal urothelium only the cell–cell borders are stained; the part of the cells in contact with the basement membrane does not react with anti-E-cadherin antibody. (**B**), (**C**), (**D**) Similar positive (normal) staining pattern in TaG1, T3aG3 and T1bG3 bladder tumours. (**E**) Heterogeneous staining in a T2G3 bladder tumour. Some cells show clear staining at the cell–cell border, whereas other areas are negative. (**F**) Negative staining in a T2G3 bladder tumour. Almost all the cells are completely negative.

The Kaplan–Meier method was used to derive the recurrence, progression and survival-free functions, while the log-rank test was used to compare curves with low and high E-cadherin expression by mean of IHC and RT-PCR. To determine the independent prognostic value of each variable, stepwise logistic regression was run using IHC group on BMDP software and odds ratios (OR) were estimated using the 95% confidence interval (CI). To compare the power and independence of each prognostic variable, we ran a multistep logistic regression procedure on BMDP software to determine (*P*) values and odd ratios (OR) in univariate and multivariate analysis.

### RESULTS

The typical pattern of immunostaining for E-Cadherin in normal urothelium and different bladder carcinomas is shown in Figure 1. In normal urothelium only the cell–cell borders were stained, as in other epithelial tissues; the part of the cells in contact with the basement membrane did not react with the anti-E-cadherin antibody (Figure 1A). Fifty tumours (45%) showed a normal staining pattern (Figure 1B, C, D), while 61 tumours (55%) showed abnormal E-cadherin expression (Figure 1E, F). Fourteen tumours (12.61%) were completely negative (Figure 1F). The prevailing

**Table 2** E-cadherin immunoreactivity according to tumour stage and grade

| Stage | Total number | Negative or heterogeneous (%) | Normal (%) | *P* value |
|---|---|---|---|---|
| Ta | 15 | 2 (13.3) | 13 (86.7) | |
| T1 | 45 | 18 (40.0) | 27 (60.0) | |
| T2 | 20 | 15 (75.0) | 5 (25.0) | |
| T3 | 22 | 19 (86.4) | 3 (13.6) | |
| T4 | 9 | 7 (77.8) | 2 (22.2) | < 0.0001 |
| Grade | | | | |
| G1 | 29 | 13 (44.8) | 16 (55.2) | |
| G2 | 32 | 9 (28.1) | 23 (71.9) | |
| G3 | 50 | 39 (78.0) | 11 (22.0) | < 0.001 |

**Table 3** Recurrence and progression as a function of stage and grade

| | Total number *n* | Recurrence *n* (%) | Progression n (%) |
|---|---|---|---|
| Stage | | | |
| Ta | 15 | 6 (40) | 1 (6.7) |
| T1 | 45 | 20 (44.4) | 10 (22.2) |
| T2 | 20 | 15 (75.0) | 12 (60) |
| T3 | 22 | 17 (77.3) | 16 (72.7) |
| T4 | 9 | 9 (100) | 9 (100) |
| Grade | | | |
| G1 | 29 | 13 (44.8) | 4 (13.8) |
| G2 | 32 | 16 (50) | 10 (31.2) |
| G3 | 50 | 38 (76.0) | 34 (68) |

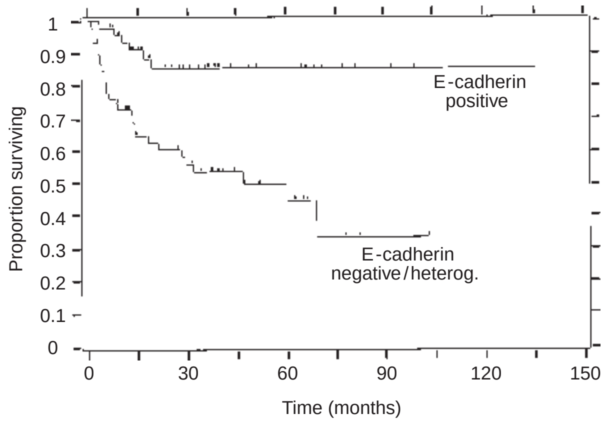

**Figure 2** Kaplan–Meier disease-specific survival curve according to E-cadherin immunostaining, E-cadherin positive vs E-cadherin negative/heterogeneous, $P = 0.0003$ log-rank test, demonstrating the effect of abnormal E-cadherin immunoreactivity on the specific survival of TCC of the bladder.

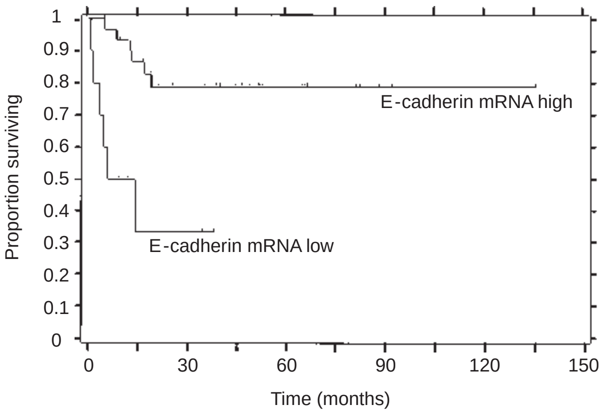

**Figure 3** Kaplan–Meier disease-specific survival curve according to E-cadherin mRNA level: high or normal level of E-cadherin mRNA vs low level of E-cadherin mRNA, $P = 0.0006$ log-rank test, demonstrating the effect of low level of E-cadherin mRNA on the specific survival of TCC of the bladder. E-cadherin mRNA levels were measured by semi-quantitative RT-PCR.

abnormal pattern was heterogeneous (47 specimens, 42.3%). Most of these specimens contained multiple foci of negative malignant cells.

Comparison of E-cadherin expression with classical histopathological features revealed that abnormal E-cadherin expression detected by immunohistochemistry correlated with both stage and grade ($P < 0.0001$ and $P < 0.001$, respectively Table 2), and with tumour shape and size ($P = 0.0007$ and $P = 0.0017$, respectively) but not with sex or multiplicity.

Analysis of recurrence, progression and cancer-specific survival (Figure 2) over a mean of 36 months after surgery in the entire cohort showed that abnormal E-cadherin immunoreactivity correlated strongly with poor outcome (log-rank test: $P = 0.001$, $P < 0.0001$ and $P = 0.0003$, respectively). Recurrence occurred in 67 patients, progression in 48 patients, relation with stage and grade is described on Table 3. The length of follow-up for surviving patients was less than 12 months for 14 patients, 12–24 months for 10 patients and more than 24 months for 49 patients. During the period study 39 patients were dying.

The level of E-cadherin mRNA correlated strongly with stage and grade, ($\chi^2$, $P = 0.0036$ and $P = 0.002$, respectively) and is also associated with poor outcome, shown in Figure 3 ($P = 0.0006$).

In univariate analysis, stage, grade, size, multiplicity and E-cadherin immunoreactivity were each found to have separate effects. However, in the final model of multistep logistic regression only E-cadherin status and stage had significant independent prognostic value ($P = 0.008$, OR = 0.2 and $P = 0.03$, OR = 3.6, respectively).

### DISCUSSION

We analysed the expression of the intercellular adhesion molecule E-cadherin in sections of normal and malignant human urothelium. In a cohort of 111 patients treated with standard protocols, E-cadherin expression was associated with tumour recurrence, progression and survival, and provided information independent of tumour stage and grade and these results were in agreement with those of Otto et al (1997).

As E-cadherin is thought to counter tumour invasion, tumours with only a minor fraction of negative cells should have invasive potential. Abnormal staining (negative or heterogeneous) was found in 20/60 (33.3%) of the superficial tumours and in 41/51 (80.4%) of the invasive tumours. This strong correlation with tumour stage indicates that altered E-cadherin expression effectively plays a role in bladder tumour invasiveness, and concords with the results of other investigations (Behrens et al, 1989; Frixen et al, 1991; Vleminckx et al, 1991).

 © 2000 Cancer Research Campaign

We also found a significant difference in E-cadherin expression according to the grade of tumour differentiation. Previous studies (Shimoyama and Hirohashi, 1991; Oka et al, 1993) showed a correlation between E-cadherin expression and the histological type of gastric and breast carcinomas. These observations are consistent with experiments in vitro suggesting that cadherins are important determinants of tissue morphology. Nagafuchi et al (1987) showed that forced expression of E-cadherin complementary DNA in fibroblastic cells generated epithelial structures, and E-cadherin causes polarized distribution of $Na^+$, $K^+$-ATPase, an important factor in establishing cell polarity (McNeill et al, 1990). E-cadherin mRNA levels was found to correlate with adverse clinical outcome (Giroldi et al, 1994; Imao et al, 1999) in accordance with the correlation of expression between immunohistochemical staining and RT-PCR as previously reported (Gil-Diez de Medina et al, 1999). These results suggest that the alteration occurs at the transcriptional level. In several cases (papillary thyroid, head and neck, colorectal and bladder cancer), E-cadherin IHC was shown to be correlated with mRNA expression (Giroldi et al, 1994; Imao et al, 1999). As no mutations or deletions have been documented in the E-cadherin gene of bladder tumours (Knowles et al, 1994; Giroldi et al, 1994), gene silencing by methylation might be involved as in prostate and breast cancer (Graff et al, 1995).

The immunohistochemical procedure is more practical than RT- PCR and can be used to assess tumour heterogeneity, which has prognostic significance. Furthermore, alterations of E-cadherin expression detected by immunohistochemistry or molecular analysis were strongly associated with grade and stage, features related to the progression of bladder cancer. These results are in line with those of Bringuier et al (1993), Lipponen and Eskelinen (1995), Ross et al (1995), Syrigos et al (1995) and Otto et al (1997), and indicate that a loss of E-cadherin expression could be one characteristic of tumour dedifferentiation.

It is striking that most tumours in our series of abnormal immunostaining (47/61) were not completely negative but were composed of positive and negative areas. Similar findings have been reported by Shiozaki et al (1991) in cancers of the oesophagus, stomach and breast, and by Bringuier et al (1993) in bladder cancer. Heterogeneous E-cadherin expression is likely to reflect the tumour heterogeneity. Mareel et al (1991) demonstrated that E-cadherin expression in Madin–Darby canine kidney cells grown in nude mice was reversibly down-regulated in vivo. Heterogeneous E-cadherin expression might thus be due not only to tumour heterogeneity but also to unstable expression in a clone in vivo.

The E-cadherin present in certain tumours, revealed by immunocytochemistry, might not be functional. For instance, certain mutations in the E-cadherin gene or changes in E-cadherin-associated cytoplasmic proteins, the catenins (Ozawa et al, 1990; Shimazui et al, 1996; Syrigos et al, 1998), may alter their adhesive functions.

The cytoplasmic tail of E-cadherin interacts with either β- or γ-catenins which bridge E-cadherin to the cytoskeleton through α-catenin. In addition, E-cadherin function is dependent on its anchoring to the cytoskeleton (Shimoyama et al, 1989; Shimazui et al, 1996). The cytoplasmic immunoreactivity found in several bladder tumours of our series might be caused by disturbed E-cadherin-cytoskeleton interactions (Shiozaki et al, 1991; Bringuier et al, 1993).

Soluble forms of E-cadherin (sE-cadherin) have been detected in the urine and serum of patients with bladder cancer (Banks et al, 1995; Griffiths et al, 1996). Concentrations of urinary E-cadherin together with increased E-cadherin amounts in the serum of some patients with higher-grade invasive tumours suggest that increased proteolytic activity in tumours may contribute to the release of the soluble form. In contrast, there was no correlation between E-cadherin immunostaining and soluble E-cadherin levels, suggesting two different mechanisms for this observation (Griffiths et al, 1996).

Tissue cadherin expression is more likely to reflect genetic abnormalities, as we found a correlation between mRNA levels and immunohistochemical staining.

Several in vitro and in vivo studies have shown that alteration or loss of E-cadherin expression is associated with a change in cell morphology, increased cell migration, and invasion (Frixen et al, 1991; Schipper et al, 1991; Vleminckx et al, 1991). In addition, positive E-cadherin expression was reported in all specimens of normal prostate, benign prostatic hyperplasia, and well-differentiated prostatic carcinoma, while expression was reduced in 90% of poorly differentiated and 93% of locally advanced prostatic carcinomas (Otto et al, 1993). It has also been reported that a decrease in E-cadherin expression correlates with poor survival in patients with bladder carcinoma (Bringuier et al, 1993; Syrigos et al, 1995; Otto et al, 1997; Imao et al, 1999).

Our results support the above findings, suggesting that E-cadherin may play an important role in the genesis of histological differentiation and affect the invasive or metastatic behaviour of bladder cancer cells in vivo. We infer that abnormal E-cadherin expression is associated with tumour recurrence, progression and survival, and has prognostic value independently of tumour stage and grade. While further investigations are required to confirm its role in cancer metastasis, our results suggest that E-cadherin expression may be used as a metastatic or prognostic marker in human bladder cancer.

The main limitation of our study is that the results were obtained on fresh frozen material. Other investigators have shown that results obtained with formalin-fixed paraffin-embedded material are not reliable (Ruijter et al, 1997). Improvement of fixation procedures in use for others molecules involved in the cell-cadherin complex, particularly catenins, have been proposed to study this biological phenomenon (Shimazui et al, 1996).

In conclusion, this study confirms the relevance of cell adhesion molecule expression to the clinical and biological behaviour of bladder cancer. This new class of marker, which is independent of clinical and pathological parameters, should now be compared to other potential markers, particularly molecules involved in cell-cycle regulations, such as Rb and p53, or cell proliferation, such as EGF-R and MIB-1.

### ACKNOWLEDGEMENTS

This study was supported in part by Institut Curie, Ligue Nationale Contre le Cancer, Comité Paris and Comité du Val de Marne (Créteil), Université Paris XII, Association Claude Bernard, Association de la Recherche Contre le Cancer, Délégation à la Recherche Clinique and Assistance Publique Hôpitaux de Paris AP-HP (PHRC No: AOA94015).

© 2000 Cancer Research Campaign

### REFERENCES

Banks RE, Porter WH, Whelan P, Smith PH and Selby PJ (1995) Soluble forms of the adhesion molecule E-cadherin in urine. *J Clin Pathol* **48**: 179–180

Behrens J (1994) Cell contacts, differentiation and invasiveness of epithelial cells. *Invasion Metastasis* **14**: 61–70

Behrens J, Mareel MM, Van Roy FM and Birchmeier W (1989) Dissecting tumor cell invasion: epithelial cells acquire invasive properties after the loss of uvomorulin-mediated cell–cell adhesion. *J Cell Biol* **108**: 2435–2447

Bringuier PP, Umbas R, Schaafsma HE, Karthaus HFM, Debruyne FMJ and Schalken JA (1993) Decreased E-cadherin immunoreactivity correlates with poor survival in patients with bladder tumors. *Cancer Res* **53**: 3241–3245

Chirgwin JM, Przybyla AE, Mac Donald RJ and Rutter WJ (1979) Isolation of biologically active ribonucleic acid from sources enriched in ribonuclease. *Biochemistry* **18**: 5294–5299

Frixen UH, Behrens J, Sachs M, Eberle G, Voss B, Warda A, Löchner D and Birchmeier W (1991) E-cadherin-mediated cell–cell adhesion prevents invasiveness of human carcinoma cells. *J Cell Biol* **113**: 173–185

Gil-Diez de Medina S, Chopin DK, El Marjou A, Delouvée A, La Rochelle WJ, Hoznek A, Abbou CC, Aaronson SA, Thiery JP and Radvanyi F (1997) Decreased expression of keratinocyte growth factor receptor in a subset of human transitional cell bladder carcinomas. *Oncogene* **14**: 323–330

Gil-Diez de Medina S, Popov Z, Chopin DK, Southgate J, Tucker GC, Delouvée A, Thiery JP and Radvanyi F (1999) Relationship between E-cadherin and fibroblast growth factor receptor 2b expression in bladder carcinomas. *Oncogene* **18**: 5722–5726

Giroldi LA, Bringuier PP and Schalken JA (1994) Defective E-cadherin function in urological cancers: clinical implications and molecular mechanisms. *Invasion Metastasis* **14**: 71–81

Giroldi LA, Bringuier PP, Shimazui T, Jansen K and Schalken JA (1999) Changes in cadherin–catenin complexes in the progression of human bladder carcinoma. *Int J Cancer* **2**: 70–76

Graff JR, Herman JG, Lapidus RG, Chopra H, Xu R, Jarrad DF, Isaacs WB, Pitha PM, Davidson NE and Baylin SB (1995) E-cadherin expression is silenced by DNA hypermethylation in human breast and prostate carcinomas. *Cancer Res* **55**: 5195–5199

Griffiths TR, Brotherick I, Bishop RI, White MD, Mc Kenna DM, Horne CW, Shenton BK, Neal DE and Mellon JK (1996) Cell adhesion molecules in bladder cancer: soluble serum E-cadherin correlates with predictors of recurrence. *Br J Cancer* **74**: 579–584

Herr HW, Badalament RA, Amato DA, Laudone VP, Fair WR Jr and Whitmore WF (1989) Superficial bladder cancer treated with bacillus Calmette–Guerin: a multivariate analysis of factors affecting tumor progression. *J Urol* **141**: 22–29

Imao T, Koshida K, Endo Y, Uchibayashi T, Sasaki T and Namiki M (1999) Dominant role of E-cadherin in the progression of bladder cancer. *J Urol* **161**: 692–698

Knowles MA, Elder PA, Williamson M, Cairns JP, Shaw ME and Law MG (1994) Allelotype of human bladder cancer. *Cancer Res* **54**: 531–538

Liotta LA, Steeg PS and Stetler-Stevenson WG (1991) Cancer metastasis and angiogenesis: an imbalance of positive and negative regulation. *Cell* **64**: 327–336

Lipponen PK and Eskelinen MJ (1995) Reduced expression of E-cadherin is related to invasive disease and frequent recurrence in bladder cancer. *J Cancer Res Clin Oncol* **121**: 303–308

Mareel MM, Behrens J, Birchmeier W, De Bruyne GK, Vleminckx K, Hoogewijs A, Fiers WC and Van Roy FM (1991) Down-regulation of E-cadherin expression in Madin–Darby canine kidney (MDCK) cells inside tumors of nude mice. *Int J Cancer* **47**: 922–928

McNeill H, Ozawa M, Kemler R and Nelson WJ (1990) Novel function of the cell adhesion molecule uvomorulin as an inducer of cell surface polarity. *Cell* **62**: 309–316

Nagafuchi A, Shirayoshi Y, Okazaki K, Yasuda K and Takeichi M (1987) Transformation of cell adhesion properties by exogenously introduced E-cadherin cDNA. *Nature* **329**: 341–343

Oka H, Shiozaki H, Kobayashi K, Inoue M, Tahara H, Kobayashi T, Takatsuka Y, Matsuyoshi N, Hirano S, Takeichi M and Mori T (1993) Expression of E-cadherin cell adhesion molecules in human breast cancer tissues and its relationship to metastasis. *Cancer Res* **53**: 1696–1701

Otto T, Rembrink K, Goepel M, Meyer Schwickerath M and Rübben H (1993) E-cadherin: a marker for differentiation and invasiveness in prostatic carcinoma. *Urol Res* **21**: 359–362

Otto T, Bex A, Schmidt U, Raz A and Rübben H (1997) Improved prognosis assessment for patients with bladder carcinoma. *J Pathol* **150**: 1919–1923

Ozawa M, Ringwald M and Kemler K (1990) Uvomorulin–catenin complex formation is regulated by a specific domain in the cytoplasmic region of the cell adhesion molecule. *Proc Natl Acad Sci USA* **87**: 4246–4250

Raghavan D, Shipley WU, Garnick MB, Russell PJ and Richie JP (1990) Biology and management of bladder cancer. *N Engl J Med* **322**: 1129–1138

Rebel JM, Thijssen CD, Vermey M, Delouvée A, Zwarthoff EC and Van Der Kwast TH (1994) E-cadherin expression determines the mode of replacement of normal urothelium by human bladder carcinoma cells. *Cancer Res* **54**: 5488–5492

Ross JS, Del Rosario AD, Figge HL, Sheehan C, Fisher H’A and Bui HX (1995) E-cadherin expression in papillary transitional cell carcinoma of the urinary bladder. *Hum Pathol* **26**: 940–944

Ruijter ET, Miller GJ, Aalders TW, Van de Kaa CA, Schalken JA, Debruyne FM and Boon ME (1997) Rapid microwave-stimulated fixation of entire prostatectomy specimens. Biomed-II MPC Study Group. *J Pathol* **183**: 369–375

Schipper JH, Frixen UH, Behrens J, Unger A, Jahnke K and Birchmeier W (1991) Ecadherin expression in squamous cell carcinomas of head and neck: inverse correlation with tumor dedifferentiation and lymph node metastasis. *Cancer Res* **51**: 6328–6337

Shimazui T, Schalken JA, Giroldi LA, Jansen CF, Akasa H, Koiso, Debruyne FM and Bringuier PP (1996) Prognostic value of cadherin-associated molecules (alpha-, beta, and gamma-catenins and p120cas) in bladder tumors. *Cancer Res* **56**: 4154–4158

Shimoyama Y and Hirohashi S (1991) Expression of E-cadherin and P-cadherin in gastric carcinomas. *Cancer Res* **51**: 2185–2192

Shimoyama Y, Hirohashi S, Hirano S, Noguchi M, Shimosato Y, Takeichi M and Abe O (1989) Cadherin cell-adhesion molecules in human epithelial tissues and carcinomas. *Cancer Res* **49**: 2128–2133

Shiozaki H, Tahara H, Oka H, Miyata M, Kobayashi K, Tamura S, Iihara K, Doki Y, Hirano S, Takeichi M and Mori T (1991) Expression of immunoreactive E-cadherin adhesion molecules in human cancers. *Am J Pathol* **139**: 17–23

Syrigos KN, Krausz T, Waxman J, Pandha H, Rowlinson-Busza G, Verne J, Epenetos AA and Pignatelli M (1995) E-cadherin expression in bladder cancer using formalin-fixed paraffin-embedded tissues: correlation with histopathological grade, tumour stage and survival. *Int J Cancer* **64**: 367–370

Syrigos KN, Harrington K, Waxman J, Krausz T and Pignatelli M (1998) Altered gamma-catenin expression correlates with poor survival in patients with bladder cancer. *J Urol* **160**: 1889–1893

Takeichi M (1991) Cadherin cell adhesion receptors as a morphogenetic regulator. *Science* **251**: 1451–1455

Takeichi M (1993) Cadherins in cancer: implications for invasion and metastasis. *Curr Opin Cell Biol* **5**: 806–811

Umbas R, Schalken JA, Aalders TW, Carter BS, Karthaus HF, Schaafsma HE, Debruyne FMJ and Isaacs WB (1992) Expression of the cellular adhesion molecule E-cadherin is reduced or absent in high-grade prostate cancer. *Cancer Res* **52**: 5104–5109

Vleminckx K, Vakaet L Jr, Mareel M, Fiers W and Van Roy F (1991) Genetic manipulation of E-cadherin expression by epithelial tumor cells reveals an invasion suppressor role. *Cell* **66**: 107–119

 *© 2000 Cancer Research Campaign*
